# The histological representativeness of glioblastoma tissue samples

**DOI:** 10.1007/s00701-020-04608-y

**Published:** 2020-10-21

**Authors:** Vilde Elisabeth Mikkelsen, Ole Solheim, Øyvind Salvesen, Sverre Helge Torp

**Affiliations:** 1 Department of Clinical and Molecular Medicine, Faculty of Medicine and Health Sciences, NTNU - Norwegian University of Science and Technology, Erling Skjalgssons gt. 1, 7491 Trondheim, Norway; 2 Department of Neuromedicine and Movement Science, Faculty of Medicine and Health Sciences, NTNU - Norwegian University of Science and Technology, Trondheim, Norway; 3 Department of Neurosurgery, St. Olavs Hospital, Trondheim University Hospital, Trondheim, Norway; 4 Department of Public Health and Nursing, Faculty of Medicine and Health Sciences, NTNU - Norwegian University of Science and Technology, Trondheim, Norway; 5 Department of Pathology, St. Olavs Hospital, Trondheim University Hospital, Trondheim, Norway

**Keywords:** Glioblastoma, Histopathology, Grading, Sampling error, Biopsy, Magnetic resonance imaging

## Abstract

**Background:**

Glioblastomas (GBMs) are known for having a vastly heterogenous histopathology. Several studies have shown that GBMs can be histologically undergraded due to sampling errors of small tissue samples. We sought to explore to what extent histological features in GBMs are dependent on the amount of viable tissue on routine slides from both biopsied and resected tumors.

**Methods:**

In 106 newly diagnosed GBM patients, we investigated associations between the presence or degree of 24 histopathological and two immunohistochemical features and the tissue amount on hematoxylin-eosin (HE) slides. The amount of viable tissue was semiquantitatively categorized as “sparse,” “medium,” or “substantial” for each case. Tissue amount was also assessed for associations with MRI volumetrics and the type of surgical procedure.

**Results:**

About half (46%) of the assessed histological and immunohistochemical features were significantly associated with tissue amount. The significant features were less present or of a lesser degree when the tissue amount was smaller. Among the significant features were most of the features relevant for diffuse astrocytic tumor grading, i.e., small necroses, palisades, microvascular proliferation, atypia, mitotic count, and Ki-67/MIB-1 proliferative index (PI).

**Conclusion:**

A substantial proportion of the assessed histological features were at risk of being underrepresented when the amount of viable tissue on HE slides was limited. Most of the grading features were dependent on tissue amount, which underlines the importance of considering sampling errors in diffuse astrocytic tumor grading. Our findings also highlight the importance of adequate tissue collection to increase the quality of diagnostics and histological research.

## Introduction

Glioblastomas (GBMs) are the most common and most malignant of the primary brain tumors in adults [30] with a median overall survival of only 10–14 months [15, 41]. The standard treatment is maximal tumor resection with adjuvant concomitant radio-chemotherapy [41].

GBMs are known for having an extensively heterogeneous histopathology [5, 24], which increases the risk of retrieving non-representative tumor samples for histological assessments. This potential for sampling errors has been demonstrated in previous studies, which have shown that GBMs can be histologically undergraded on biopsies [4, 8, 11, 16, 25, 26, 29, 36, 45]. The GBM diagnosis is today based on both histological and molecular analyses according to the World Health Organization’s (WHO) Classification of Tumors of the Central Nervous System [24]. Here, GBMs are histologically classified as diffuse astrocytomas of the highest malignancy grade (i.e., diffuse astrocytoma grade IV) [24]. The grading is based on the presence of the histopathological features atypia, mitotic activity, increased cellular density, microvascular proliferation, and necrosis. The presence of either of the latter two is mandatory for the grade IV. In 2016, the mutation status of the isocitrate dehydrogenase (IDH) enzyme was implemented in the WHO classification, where it diagnostically stratifies the GBMs into IDH wildtype (wt) and IDH mutant (mt) [24]. Recently, extensive molecular analyses such as methylation profiling have been shown as promising tools in improving the diagnostic accuracy of brain tumors [6, 7, 18]. However, these comprehensive molecular analyses are not yet available to many institutions [2, 37]. Hence, the risk of retrieving non-representative histological samples is a highly relevant limitation in glioma diagnostics and research.

Previous studies have found a correlation between a smaller volume of the pathological specimens and a lower rate of GBM diagnosis [12, 19]. However, to our knowledge, no previous studies have investigated relationships between the amount of viable tissue on hematoxylin-eosin (HE) slides and the presence of individual histological features in GBMs. We therefore aimed to explore to what extent the histology of GBMs is affected by tissue amount by investigating associations between subjectively assessed area of viable tissue on HE slides and the presence or degree of 24 histopathological features and immunohistochemical quantifications of Ki-67/MIB-1 (proliferative index (PI)) and CD105/endoglin (microvessel density (MVD)). In addition, we assessed associations between the tissue amount and MRI volumetrics, the type of surgical procedure, the number of HE slides, and estimated tissue volumes.

## Material and methods

### Inclusion and exclusion criteria

The inclusion of the 106 patients is based on the previous work by Stensjøen et al. [39] where the preoperative growth dynamics of GMBs were explored. The patients were retrospectively selected from 262 consecutive patients ≥ 18 years with newly diagnosed GBMs operated at St. Olavs Hospital, Trondheim University Hospital, Norway, between January 2004 and May 2014. Selection criteria were (i) ≥ 2 preoperative T_1_-weighted contrast (gadolinium) enhancing (T_1_wGd) magnetic resonance imaging (MRI) scans taken ≥ 14 days apart and (ii) histopathologically verified diagnosis after the 2016 WHO classification [24]. Exclusion criteria were (i) gliomatosis cerebri and (ii) non-contrast-enhancing tumors. The IDH mutation status has previously been assessed, first with immunohistochemistry for IDH-R132H [40], and all immunonegative patients < 55 years had additional Sanger sequencing of IDH1/2 according to previously described methods [17]. Patients that had inadequate IDH2 sequencing but were wildtype on IDH1 sequencing were categorized as IDH wt due to the very low frequency of IDH2 mutations in GBMs [3, 20]. We did not exclude IDH mt and not otherwise specified (NOS) cases due to their similar histopathology to IDH wt GBMs [24]. Clinical data, such as the type of surgical procedure, have previously been collected and accounted for [40]. Total tumor volumes and volumes of the contrast-enhancing compartment have previously been segmented from the preoperative T_1_wGd MRI scans (taken for intraoperative neuronavigation) [39]. Total tumor volume was defined as the combination of the contrast-enhancing rim and the non-contrast-enhancing (necrotic) core [39].

### Quantification of tissue amount

**Fig. 1:**
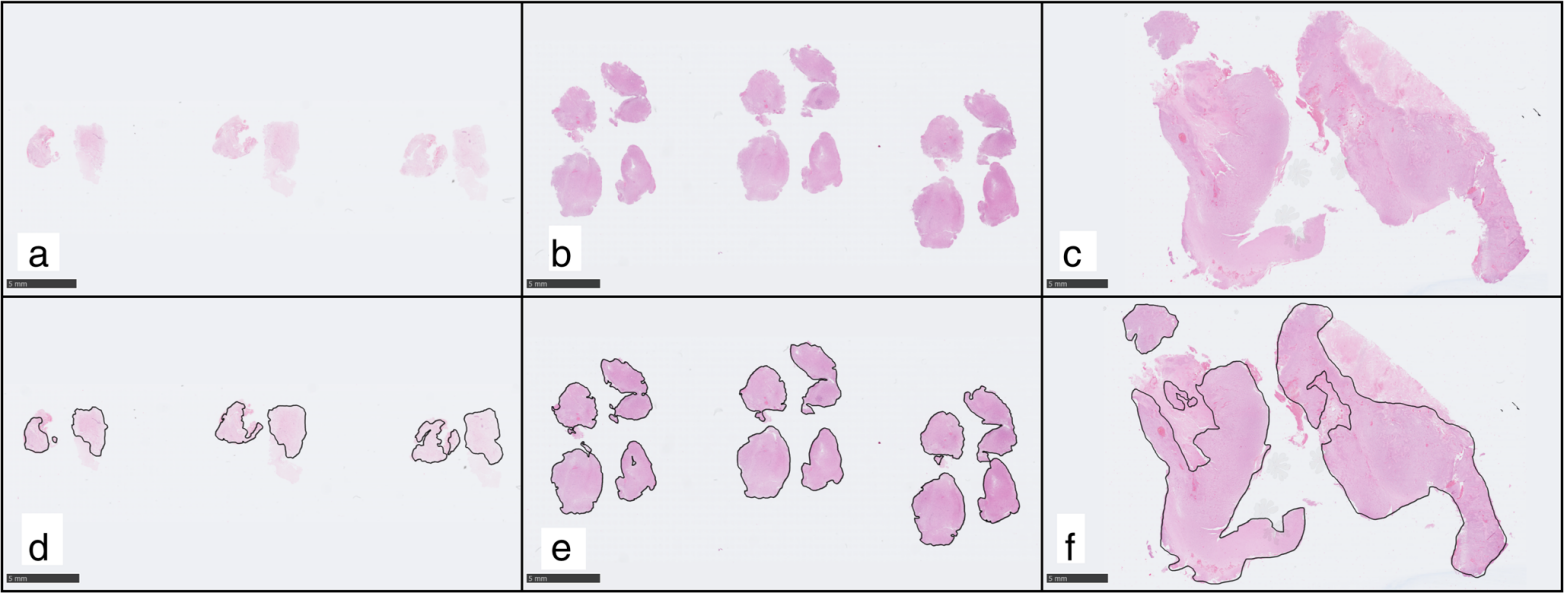
Examples of the three categories of tissue amount. The area of viable tissue was subjectively categorized in each case into the categories “sparse” (**a**, **d**), “medium” (**b**, **e**), or “substantial” (**c**, **f**). **d**–**f** Annotations of the viable proportion of the tissue in the same cases (**a**–**c**). In these examples, the collective areas of viable tissue were 32 mm^2^ for the “sparse” category (**d**), 110 mm^2^ for the “medium” category (**e**), and 279 mm^2^ for the “substantial” category (**f**). All three examples had no additional routine HE slides. The “sparse” example had no additional FFPE slides from previously frozen tissue, whereas both the “medium” and “substantial” example had one additional section from previously frozen tissue with sparse tissue amount. All three exemplified cases had resections. Hematoxylin-eosin stained tissue slides at × 0.5 magnification. Scale bars 5 mm. Tissue slides were scanned with a Hamamatsu NanoZoomer S60 scanner (Hamamatsu Photonics, Japan) and images created from exportations using the NDP.view2 software (version 2.7.52) (Hamamatsu)

Tissue amount was subjectively quantified as the combined area of viable (i.e., non-necrotic) tissue on all available HE slides retrieved from the first surgical intervention in each patient (including slides from previously frozen formalin-fixed paraffin-embedded (FFPE) tissue). The area was semiquantitatively categorized as “sparse,” “medium,” or “substantial,” and Fig. 1 illustrates examples from each tissue category. The number of slides (i.e., the number of tissue blocks) was recorded in each patient. One patient had only sections from previously frozen FFPE tissue, and 8 cases had no additional slides from frozen FFPE tissue. Sections from previously frozen FFPE tissue generally had quite small areas of viable tumor that contributed to a minor degree to the total amount. We also estimated the tissue volume (cm^3^) in each case from the diameter of the tissue samples sent for neuropathology, using the formula of an ellipsoid volume described by Gutt-Will et al. [12].

### Histopathology and immunohistochemistry

The registration of the 24 assessed histopathological features listed in Table 2 was performed in a previous study [27], which contains detailed definitions of each of the features. All HE slides from each case (both from routine and previously frozen FFPE tissue) were investigated for the presence or the degree of the 24 histopathological features and two immunohistochemical features. Cellular density and atypia were semiquantitatively graded into 3 categories [27]. Mitoses were counted in hotspots from 10 high power fields (HPFs) at × 400 magnification [27].

The immunohistochemical procedures for the staining of the proliferative marker Ki67/MIB-1 (monoclonal, Ki-67/MIB-1, 1:800 or 1:50, Dako, Glostrup, Denmark) and the endothelial marker CD105/endoglin (monoclonal, CD105/endoglin/SN6h, 1:50, Dako) have previously been accounted for [28, 40]. The proliferative index (PI) of Ki67/MIB-1 was quantified as the percentage of distinctly positive tumor cells in hotspots in three HPFs, as previously described [40]. In another previous work, we quantified the microvessel densities (MVDs) of CD105 and vWF as the mean number of positively staining vascular units in hotspots in three HPFs at × 400 magnification using an eyepiece grid [28]. We only included CD105-MVD in the current study, because the MVDs were highly correlated and only CD105-MVD was significantly associated with radiological tumor growth [28].

### Statistical analyses

Statistical analyses were performed using Stata version 16 and the limit of statistical significance set to *p* ≤ 0.05. Associations between the three categories of tissue amount and categorical variables were assessed using chi-square/Fisher’s exact tests, and associations with quantitative variables were assessed using Kruskal-Wallis tests. In the crosstab analyses, *p* values were recorded from the Fisher’s exact test when ≥ 1 of the expected values were ≤ 5. The significant variables in the Kruskal-Wallis tests were tested for post hoc pairwise comparisons using Mann-Whitney *U* tests between subgroups.

## Results

### Patient characteristics

Thirty-two percent of the patients were female (34 patients), and the mean age at diagnosis was 63 years, range 26–83. Three patients were IDH mt, one was IDH NOS, and the rest were IDH wt. Six of the IDH wt cases had inconclusive results from the IDH2 sequencing but were wildtype on the IDH1 sequencing. All cases were immunohistochemically positive for glial fibrillary acidic protein (GFAP). The median number of HE slides (i.e., the number of tissue blocks) per patient was 3 (range 1–23); this number included both routine sections (median 1, range 0–23) and sections from previously frozen FFPE tissue (median 1, range 0–5). The median total tissue volume was 0.74 cm^3^ (range 0.02–42.85), which included routine FFPE tissue (median 0.54 cm^3^, range 0.01–42.75) and previously frozen FFPE tissue (0.11 cm^3^, range 0.00–6.84).

Distributions of the type of surgical procedure, MRI volumetrics, the number of HE slides, and the tissue volumes across the tissue amount categories are presented in Table 1. Type of surgical procedure, i.e.; biopsy or resection, was significantly associated with tissue amount. Most of the biopsied cases had “sparse” tissue amount (88%); however, most cases (69%) with “sparse” tissue amount were specimens from resections (cytoreduction or gross total resection (GTR)). Tissue amount was not significantly associated with any of the MRI volumetrics, but it was strongly associated with the number of HE slides and estimated tissue volume (Tables 1 and 3).

**Table 1:** Clinical features and tissue amount

	Sparse tissue (*n* = 49)	Medium tissue (*n* = 29)	Substantial tissue (*n* = 28)	*p* value	Test performed
Surgical procedure					
• Biopsy (*n*)	31% (15)	7% (2)	0% (0)		Fisher’s exact
• Cytoreduction (*n*)	43% (21)	66% (19)	68% (19)		
• GTR (*n*)	27% (13)	28% (8)	32% (9)	0.003*	
Median preoperative total tumor volume (range)	24.0 mL (1.7–92.9)	33.1 mL (1.0–82.9)	35.5 mL (1.5–243.5)	0.176	Kruskal-Wallis
Median preoperative contrast-enhancing volume (range)	15.1 mL (1.0–53.4)	15.7 mL (0.9–63.9)	23.2 mL (1.4–215.4)	0.246	Kruskal-Wallis
Median number of tissue sections (range)	2 (1–18)	2 (2–19)	4 (2–23)	< 0.001*	Kruskal-Wallis
Median tissue volume (range)^a^	0.13 cm^3^ (0.02–3.66)	1.36 cm^3^ (0.06–40.74)	11.63 cm^3^ (2.88–42.85)	< 0.001*	Kruskal-Wallis

*GTR* gross total resection, *CI* confidence interval, *n* absolute number of cases

^a^Estimated in 89 of the patients, tissue volume was not possible to estimate in the excluded cases due to inadequate descriptions

*Statistically significant, *p* ≤ 0.05

Distributions of the type of surgical procedure and preoperative MRI volumetrics across the tissue amount categories. The *p* values are from tests of association between the clinical features and tissue amount

### Histopathology and tissue amount

Distributions of the 24 histological and the two immunohistochemical features within the categories of tissue amount are shown in Table 2. The features significantly associated with tissue amount were small necroses, palisades, microvascular proliferation, atypia, mitotic count, hemorrhages, pseudorosettes, subpial clustering, lymphocytic infiltration, small cell differentiation, Ki-67/MIB-1 PI, and CD105-MVD (Table 2). All significant dichotomous features were less present in cases with “sparse” tissue amount. Atypia tended to be more severe in cases with more available tissue, and only cases with “sparse” tissue amount were categorized as “mild” atypia. For the quantitative variables mitotic count, Ki-67/MIB-1 PI, and CD105-MVD, pairwise comparisons of subgroups are found in Table 3. Mitotic count and Ki-67/MIB-1 PI were both significantly lower in the “sparse” versus “medium” tissue category, whereas the CD105-MVD counts were significantly higher in the “substantial” category than the two other categories (Table 3).

**Table 2:** Histopathology and tissue amount

Histopathological feature	Sparse tissue (*n* = 49)	Medium tissue (*n* = 29)	Substantial tissue (*n* = 28)	*p* value	Test performed
Necroses					
• Large, ischemic (*n*)	90% (44)	90% (26)	89% (25)	1.000	Fisher’s exact
• Small (*n*)	69% (34)	90% (26)	100% (28)	0.001*	Fisher’s exact
Palisades (*n*)^a^	51% (25)	86% (24)	89% (25)	< 0.001*	Chi-square
Microvascular proliferation (*n*)	55% (27)	90% (26)	100% (28)	< 0.001*	Chi-square
Cellular density					
• Low (*n*)	10% (5)	0% (0)	0% (0)		Fisher’s exact
• Moderate (*n*)	69% (34)	59% (17)	64% (18)		
• High (*n*)	20% (10)	41% (12)	36% (10)	0.074	
Atypia					
• Mild (*n*)	6% (3)	0% (0)	0% (0)		Fisher’s exact
• Moderate (*n*)	84% (41)	79% (23)	61% (17)		
• Severe (*n*)	10% (5)	21% (6)	39% (11)	0.017*	
Median mitotic count (range)^b^	5 (0–34)	13 (0–65)	22 (2–43)	< 0.001*	Kruskal-Wallis
Vascular features					
• Thromboses (*n*)	78% (38)	83% (24)	93% (26)	0.280	Fisher’s exact
• Hemorrhage (*n*)	67% (33)	86% (25)	93% (26)	0.016*	Chi-square
• Pseudorosettes (*n*)^c^	9% (4)	25% (7)	39% (11)	0.006*	Chi-square
Secondary structures of Scherer					
• Perineuronal satellitosis (*n*)^d^	52% (13)	43% (6)	52% (14)	0.834	Chi-square
• Angiocentric structures (*n*)^d^	32% (8)	50% (7)	44% (12)	0.487	Chi-square
• Subpial clustering (*n*)^e^	0% (0)	40% (4)	33% (8)	0.042*	Fisher’s exact
Desmoplasia (*n*)	57% (28)	66% (19)	71% (20)	0.437	Chi-square
Leukocytes					
• Macrophages (*n*)	90% (44)	93% (27)	100% (28)	0.314	Fisher’s exact
• Lymphocytic infiltration (*n*)	53% (26)	62% (18)	86% (24)	0.015*	Chi-square
Small cell glioblastoma (*n*)	8% (4)	14% (4)	29% (8)	0.057	Fisher’s exact
Cellular differentiation					
• Gemistocytes (*n*)	14% (7)	31% (9)	25% (7)	0.197	Chi-square
• Small cells (*n*)	12% (6)	41% (12)	11% (3)	0.003*	Chi-square
• Sarcomatous cells (*n*)	16% (8)	17% (5)	21% (6)	0.849	Chi-square
• Myxomatoid (*n*)	14% (7)	14% (4)	11% (3)	0.939	Fisher’s exact
• Giant cells (*n*)	8% (4)	7% (2)	14% (4)	0.631	Fisher’s exact
• Primitive neuronal (*n*)	6% (3)	14% (4)	4% (1)	0.400	Fisher’s exact
• Oligodendroglial (*n*)	4% (2)	10% (3)	7% (2)	0.541	Fisher’s exact
Median Ki67/MIB-1 PI (range)	11.5 (1.4–57.3)	17.5 (5.3–53.3)	14.7 (5.1–37.3)	0.036*	Kruskal-Wallis
Median CD105-MVD count (range)^f^	10.3 (0.7–48)	12.7 (6–37.7)	18.3 (1.7–50)	0.003*	Kruskal-Wallis

*PI* proliferative index, *MVD* microvessel density, *n* absolute number of cases

^a^One case was not possible to assess for palisades

^b^One case had inadequate morphology for the counting of mitoses

^c^Three cases could not be assessed for pseudorosettes

^d^Only cases with infiltration zones into gray matter were assessed (*n* = 66) [27]

^e^Only cases showing areas with outer brain surface were assessed (*n* = 45) [27]

^f^Five cases could not be assessed for CD105-MVD [28]

*Significantly associated, *p* ≤ 0.05

Distributions of the number of cases or median values of the histological features within the tissue amount categories. The* p* values are from tests of association between histology and tissue amount

**Table 3:** Post hoc pairwise comparisons of the tissue amount subgroups and quantitative variables

Tissue amount	“Sparse” vs “medium”	“Sparse” vs “substantial”	“Medium” vs “substantial”
Mitotic count	0.002*	< 0.001*	0.198
Ki-67/MIB-1 PI	0.018*	0.071	0.555
CD105-MVD	0.088	0.001*	0.033*
Number of sections	0.394	< 0.001*	< 0.001*
Tissue volume	< 0.001*	< 0.001*	< 0.001*

*PI* proliferative index, *MVD* microvessel density, *vs* versus

*Significant associations, *p* ≤ 0.05

The table presents *p* values from subgroup Mann-Whitney *U* analyses of association between quantitative variables and the tissue categories

## Discussion

We found that a substantial proportion (46%) of the assessed histopathological and immunohistochemical features were significantly associated with the amount of viable tumor material on HE slides. All significantly associated features were found to be less present or of a lesser degree in cases with a smaller amount of tissue. Several of the significant features are relevant for the grading of diffuse astrocytic tumors, i.e., small necroses, palisades, microvascular proliferation, atypia, mitotic count, and Ki-67/MIB-1 PI. We also found that “sparse” tissue amount was strongly associated with a smaller tissue volume sent for neuropathology, indicating that neurosurgical sampling impacts the histology. Interestingly, “sparse” tissue amount was commonly obtained from surgical resections, where it presumably would be possible to provide larger or more tumor samples. Our results show that several of the histopathological features in GBMs are heterogeneously distributed, which limits the histological representativeness of small tissue samples. These findings underline the importance of adequate tissue collection to increase diagnostic accuracy and quality of histological research.

Previous studies have demonstrated the risk of histological undergrading of GBMs on small tissue samples [4, 8, 11, 12, 16, 19, 25, 26, 29, 36, 45]. In contrast to our study, these studies were focused on grading, whereas our study assessed the representativeness of individual histological features. These previous studies also only focused on biopsied cases [4, 8, 11, 16, 25, 26, 29, 36, 45] or on the volume of the pathological specimen sent for analysis [12, 19]. Hence, the role of sampling errors in resected tumors is less studied. Moreover, none of the previous studies accounted for the presence of necrosis in the material, which is likely to cause a further decrease of the histological representativeness. As most of the histological features assessed in this study are only found in the viable tumor tissue, their representativeness is more precisely estimated by quantifying the area of the *viable* tumor as the tissue amount.

### Grading features

Several of the hallmark features of GBMs, small necroses, palisades, and microvascular proliferation, were significantly less present in cases with “sparse” tumor material. The significant associations suggest that these features are heterogeneously distributed, which limit their representativeness in small tissue samples. The only hallmark feature that was not significantly dependent on tissue amount was large, ischemic necrosis. This feature was found in a high proportion in all tissue categories, suggesting that it is a very frequent and homogenously distributed feature in GBMs. The fact that 90% of the cases (44 cases) with “sparse” material had large necrosis indicates that most of these cases were never at risk of being undergraded despite the scant amount of viable tissue. However, the diagnosis of the five remaining cases without large necrosis relied solely on the presence of the other hallmark features shown to be at risk of underrepresentation. Still, all five had visible necrosis on the preoperative MRI scan (data not shown) and would therefore have been treated as GBMs by many institutions, because it has been shown that lower grade astrocytomas with radiological necrosis exhibit comparable survival to GBMs [22]. Hence, our study highlights the importance of considering clinical and neuroradiological information in glioma diagnostics due to the risk of histological undergrading of small tissue samples.

In addition to the hallmark features, other features relevant for grading of diffuse astrocytic tumors, mitotic count, atypia, and Ki-67/MIB-1 PI, were also significantly associated with tissue amount. Cellular density was not significantly associated; however, there was a near-significant trend that cases with “sparse” tissue were more often categorized as “mild” and less often as “high.” Both mitotic count and Ki-67/MIB-1 PI were significantly higher in the “medium” versus “sparse” tissue categories, but neither was significantly different between the “medium” and the “substantial” categories. These findings are in accordance with the known regional heterogeneity of proliferative cells [9, 32, 33] and highlight the limitation of sampling errors in proliferative quantifications of GBMs. Interestingly, both atypia and cellular density were only categorized as “low” or “mild” in cases with “sparse” tissue amount, which suggests that these “sparse” samples might have been taken from infiltration zones of the tumor. Our findings are in line with the previous studies showing that GBMs can be histologically undergraded on small tissue samples [4, 8, 11, 16, 19, 25, 26, 29, 36, 45]. Moreover, it is also likely that some IDH wt grade II and III tumors with molecular features of GBM represent undergraded IDH wt GBMs [7, 37], as it has been shown that these tumors follow the same clinical course as GBMs [2, 37, 43]. However, undergrading is a less probable cause when radiology is in accordance with low-grade glioma [14, 43], and it has been suggested that such tumors may represent early stage GBMs [14]. Nevertheless, our study is in line with studies indicating that some of the IDH wt diffuse astrocytic gliomas with molecular features of GBM are undergraded IDH wt GBMs.

In this study, we did not assess other molecular parameters than IDH mutation status. However, as mentioned, extensive molecular analyses such as next-generation sequencing and methylation profiling have been shown to be useful tools in glioma diagnostics [6, 38]. Especially methylation profiling in combination with standard histopathology has shown promising results [6, 7, 18]. Two prospective studies showed that the use of methylation profiling led to a change in diagnosis in 12% of cases [6] and in 84% of diagnostically challenging cases [18]. The latter study also found a substantial clinical benefit of the change in diagnosis [18]. Unfortunately, intratumoral heterogeneity is also a limitation of the molecular analyses, as studies have found that different molecular GBM subtypes can exist within the same tumor [31, 47]. However, despite the finding of varying methylation subtypes, all spatially collected biopsies from the same tumor were consistently classified as GBM IDH wt or mt [47]. Still, methylation profiling is limited when tumor material is scant, illustrated by a large study in which 4% of the patients could not be profiled due to a low tumor cell content [6]. Other limitations of methylation profiling are the long turnaround time (a median of 25 days in one trial) [18], and that it is not available to most centers [2, 37]. Therefore, despite the promising introduction of extensive molecular analyses in glioma grading, the limitation of reduced histological representativeness of small tissue samples is still highly relevant.

### Other features

In addition to the abovementioned grading features, hemorrhages, pseudorosettes, subpial clustering, lymphocytic infiltration, small cells, and CD105-MVD were also significantly associated with tissue material. All the features except CD105-MVD were significantly less present in cases with “sparse” material, suggesting that these are heterogeneously distributed features. Regarding CD105-MVD, it was only significantly higher in the “substantial” tissue category than in the two lower categories, which suggests a large degree of heterogeneity in the distribution of vascular hotspots. Despite the well-known observed heterogeneity in the vascular structures on GBMs [35, 46], the degree of the heterogeneity has been sparsely studied. However, in accordance with our findings, Di Ieva et al. [10] found a large degree of heterogeneity of the vascularity of GBMs measured by digital pathology.

Thrombosis, perineuronal satellitosis, angiocentric structures, desmoplasia, macrophages, and all the cellular differentiation patterns despite small cells were not significantly associated with tissue amount. The findings indicate that these features are homogenously distributed and less prone to sampling errors. Consequently, these features have potential clinical utility in that their presence could suggest a grade IV diagnosis, given that the features have been found to strongly associate with a GBM diagnosis. Thrombosis is of particular interest, as it has been shown to associate with aggressiveness in diffuse astrocytic tumors [1, 44] and it has been suggested as a diagnostic criterion of GBM [34, 42, 44]. One study also found that the presence of thrombosis independently predicted wildtype IDH status, and they therefore suggested screening for thromboses in IDH1-R132H-negative lesions to help decide if additional sequencing of IDH1/2 is worthwhile when resources are limited [44]. Like thromboses, macrophages have been associated with aggressiveness in gliomas, and the number of macrophages has been found to increase with higher astrocytoma grades [13, 21]. However, we only recorded distinct macrophages in HE sections (i.e., not immunostained), which predominately were foamy macrophages found at the edge of necroses. Hence, the high frequency of macrophages is probably explained by the widespread presence of necrosis, and the clinical utility of macrophages is therefore limited. Moreover, the clinical utility of the secondary structures of Scherer is limited by their frequent presence in lower grade diffuse astrocytic tumors [23]. Regarding desmoplasia and the cellular differentiation patters, these are epiphenomena of the aggressive GBM biology. However, these features can also be found in other lower grade gliomas that are relevant differential diagnoses [24]. In summary, of the non-significant features, only thromboses have promising clinical utility in that their presence in a histologically lower grade IDH wt tumors could indicate that it is an undersampled IDH wt GBM.

### Clinical features

Perhaps to no surprise, the tissue amount was significantly associated with the type of surgical procedure, the number of HE slides, and tissue volume. However, the tissue amount was not associated with either total tumor volumes or volumes of the contrast-enhancing compartment on the preoperative T1wGd MRI scans, which suggest that larger tumors and more contrast enhancement did not impact histology. On the other hand, the strong association between tissue amount and tissue volume indicates that neurosurgical sampling affects the histopathology. The same association was also found when biopsied cases were excluded (*p* < 0.001, data not shown). Put together with the finding that most of the cases with “sparse” tissue had undergone resections, our data suggest that more tissue could have been retrieved from the resected tumors. Our findings are in agreement with the study by Lasocki et al. [22], which showed that undergrading also occurred in patients who had resections. Extensive necrosis can also cause a smaller amount of viable tissue, and it is likely the explanation for the relatively large tissue volumes found in the upper range in “sparse” and “medium” tissue categories. Nevertheless, our findings indicate that neurosurgeons should be encouraged to send larger tumor samples to the pathologist to avoid potential histological undergrading.

### Strengths and limitations

The main strength of this study is the relatively large number of patients with preoperative MRI scans. The age and sex distributions were not significantly different from either the excluded or the general GBM patients in Norway [39]. Important limitations are interobserver variability of the histopathology and the subjective assessment of tissue amount. The estimation of tissue volumes was limited by a varying quality of the descriptions of the tissue diameter and that only one diameter of the tissue was typically recorded. Despite multiple statistical tests, we chose not to correct for multiple comparisons. As many as 46% of the analyses of histology and tissue amount were significant, and a couple of these are therefore likely false-positive findings. Still, the high percentage relative to the statistical limit of 5% indicates that most of these associations are true positive findings, which further substantiates our finding that the histopathological representativeness is reduced in small tissue samples of GBMs.

## Conclusion

Our study highlights the limited histological representativeness of small tissue samples of GBMs in both biopsied and resected tumors. A substantial proportion of the assessed histological features were at risk of being underrepresented when tissue material was limited, including most of the grading features. These findings underline the importance of considering sampling errors in the grading of diffuse astrocytic tumors and encourage neurosurgeons to send larger tumor samples to increase quality of diagnostics and histological research.

## Abbreviations

FFPE: Formalin-fixed paraffin-embedded
GBM: Glioblastoma
GFAP: Glial fibrillary acidic protein
GTR: Gross total resection
HE: Hematoxylin-eosin
HPF: High power field
IDH: Isocitrate dehydrogenase
MRI: Magnetic resonance imaging
MVD: Microvessel density
NOS: Not otherwise specified
PI: Proliferative index
T_1_wGd: T_1_-weighted contrast (gadolinium) enhancing
vWF: von Willebrand factor
